# Navigating Penile Metastases: Personalized Management in a Rare Oncologic Challenge

**DOI:** 10.1155/criu/7231390

**Published:** 2025-10-03

**Authors:** R. Hamal, A. Feyaerts, S. Thiry, H. Dano, J. Van Damme

**Affiliations:** ^1^Department of Urology, University Hospital Saint-Luc, Brussels, Belgium; ^2^Department of Pathology, University Hospital Saint-Luc, Brussels, Belgium

**Keywords:** cancer, metastasis, penile, renal cell carcinoma

## Abstract

Secondary malignancy of the penis is a rare clinical entity. Nearly three-quarters of metastatic lesions in the penis originate from genitourinary and pelvic organs (such as the bladder, prostate, and colon). Less than 25% of penile metastases arise from extrapelvic primary sites and are usually associated with disseminated disease. Fewer than 50 cases of renal cancer metastasis to the penis have been described in the literature to date.

We present the case of a 47-year-old male patient with a single metastasis of papillary renal cell carcinoma (pRCC) in the left corpus cavernosum. The patient had a history of left radical nephrectomy in 2004 and two lung wedge resections for unique metastases in 2007 and 2009. The patient complained of a growing, painless mass at the base of his penis. No other sites of metastasis were identified at staging. We performed a complete excision of the mass, and the final histopathological report confirmed the metastasis of pRCC with negative surgical margins. The patient remains treatment-free 8 years later.

## 1. Introduction

Renal cancer accounts for 2%–3% of all cancers in Western countries. There is a male predominance of 1.5:1, and the median age of diagnosis is between 60 and 70 years [1, 2].

Three histological types of renal cell carcinoma exist, ordered by frequency: clear cell renal cell carcinoma (ccRCC), papillary renal cell carcinoma (pRCC), and chromophobe renal cell carcinoma (cRCC).

pRCC is more likely to be localized at the time of diagnosis compared to ccRCC. pRCC has a better 5-year survival rate when confined to the kidney; however, it has a poor prognosis when metastatic [3]. Only 1.7% of pRCC patients present with metastasis at diagnosis [4].

Renal cell carcinoma metastasizes preferentially to the lung, liver, bones, brain, and adrenal glands. An extremely rare presentation is penile metastasis.

Secondary malignancy of the penis is a rare clinical entity. In 75% of cases, metastatic lesions originate from pelvic organs, primarily bladder, prostate, and colon. Less than 25% of penile metastases originate from extrapelvic primary sites. These cases are typically associated with widespread disease and are therefore linked to a poorer prognosis.” [5] Clinical manifestations of penile metastases typically present as a penile mass, skin lesion, or malignant priapism. Treatment may be systemic, local, or a combination of both, depending on the primary tumor. We present the case of a patient with a unique metastasis in the left corpus cavernosum from a pRCC, occurring 13 years after a left radical nephrectomy.

## 2. Case Report

A 47-year-old patient with metastatic pRCC underwent a left radical nephrectomy in 2004, followed by two pulmonary wedge resections: one on the left in 2007 and one on the right in 2009.

**Figure 1:**
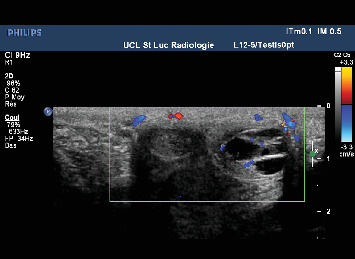
Doppler ultrasound of the penis: heterogeneous and oval formation measuring 15 × 9 mm with peripheral and septal vascularisation inside the left corpus cavernosum.

**Figure 2:**
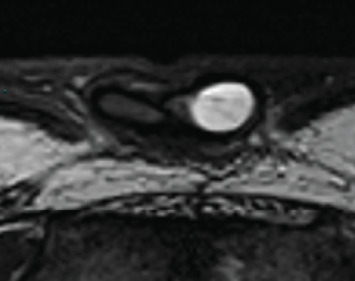
MRI of the penis: a well-circumscribed multilocular cystic structure with enhancement of the septa located at the laterodorsal part of the base of the left corpus cavernosum.

In July 2017, he complained of a palpable nodule at the base of his penis. Clinical examination revealed a 2-cm circular mass, slightly painful, at the base of the left corpus cavernosum. A Doppler ultrasound of the penis showed a heterogeneous (liquid/solid) oval formation measuring 15 × 9 **mm** with peripheral and septal vascularization inside the left corpus cavernosum (Figure 1). An MRI of the penis confirmed a well-circumscribed multilocular cystic structure with enhancement of the septa located at the laterodorsal part of the base of the left corpus cavernosum (Figure 2). A surgical excision was decided upon. No other sites of metastasis were identified during staging.

**Figure 3:**
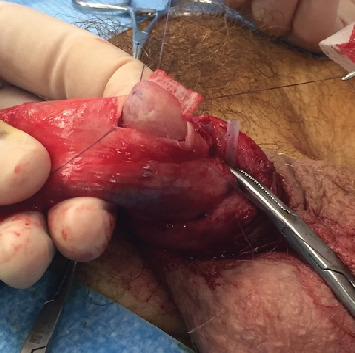
Dissection of the tumor.

**Figure 4:**
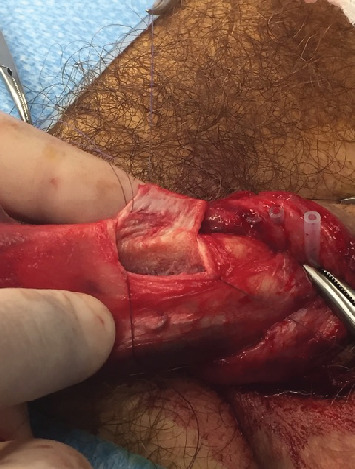
Extraction of the tumor.

**Figure 5:**
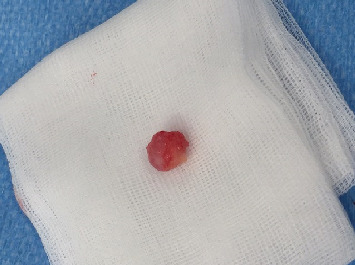
Extracted tumor without effraction.

Surgery was performed in September 2017. The patient was positioned on his back, and a Ch16 bladder catheter was inserted without resistance. Degloving of the penis allowed easy tracking of the mass. A tourniquet was placed below the lesion. The tunica albuginea facing the lesion was opened with a cold knife. The tumor was easily dissected and extracted en bloc (Figure 3), leaving a cavity in the left corpus cavernosum (Figures 4 and 5). The tunica albuginea was closed with absorbable sutures. Frozen section analysis confirmed a probable adenocarcinoma with negative surgical margins. No provoked erectile test was performed intraoperatively.

The bladder catheter was removed on Day 2, and the patient was discharged from the hospital with analgesia.

**Figure 6:**
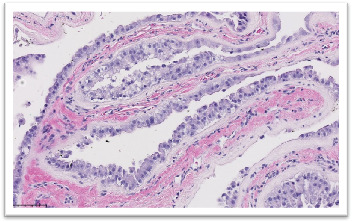
H&E section showing pseudopapillary structure composed of cells with eosinophilic cytoplasm and moderately atypical nuclei.

The final pathology report concluded that the lesion was a metastasis of pRCC. The histological findings were compatible with a Type 2 papillary carcinoma (Figure 6). Immunohistochemistry markers (CK broad spectrum, PAX8, AMACR, CD10, and CK7) confirmed the diagnosis (Table 1). Seven years after treatment, the patient remains alive with no local recurrence. No additional treatments were given, but a few pulmonary nodules are being monitored.

## 3. Discussion

Penile metastases from RCC are extremely rare. We found only one case report in the literature of a penile metastasis originating from pRCC. Secondary penile malignancies commonly arise from urogenital cancers (such as prostatic adenocarcinoma and urothelial carcinoma) or gastrointestinal cancers. In rare cases, pulmonary, hematopoietic, or cutaneous cancers can also metastasize to the penis.

These metastases can affect various anatomical regions of the penis: shaft, glans, and/or corpus cavernosum. In all cases of shaft invasion, the corpora cavernosa were also involved [6]. Our patient presented with an intracavernous nodule without signs of shaft invasion.

A 2010 study reported a mean interval of 16 months between the primary tumor and penile metastasis [6]. In our patient, the interval exceeded 13 years, with no pain or priapism. Penile metastases typically present as a palpable mass and/or malignant priapism [7].

Several hypotheses for the dissemination of malignant cells to the penis have been proposed, including retrograde venous route, lymphatic route, arterial spread, local extension, and dissemination after instrumentation.

Retrograde venous dissemination is the most likely mechanism for penile metastasis due to the anatomy of the male genital venous system. Anatomical communications between the dorsal penile venous system and the pelvic venous plexuses allow retrograde flow in the event of obstruction, facilitating the dissemination of malignant cell. This explains why secondary lesions are most commonly found in the corpus cavernosum and glans of the penis. Lymph nodes draining the penis, bladder base, posterior prostate, and lower rectum facilitate tumor spread via vessels, either through permeation or as emboli. This mechanism explains why lymphatic metastases occur more commonly in the penile skin rather than in the corpus cavernosum or glans. Arterial dissemination is less common but may explain extrapelvic metastasis, as cancer cells can become trapped in capillaries or venous plexuses, as in this case.

Medical imaging plays a central role in assessing and diagnosing primary and secondary penile tumors [8, 9]. Doppler ultrasonography allows for the examination of the penis and inguinal lymph nodes. Improved high-frequency probes have made this technique more effective. Ultrasonography typically highlights nodules of varying echogenicity and vascularization and can identify diffuse or focal infiltration of the tunica albuginea or irregular bulking of the shaft. Magnetic resonance imaging (MRI) offers a detailed view of penile anatomy. Metastatic lesions often involve multiple levels of the corpus cavernosum or spongiosum, distinguishing them from primary lesions. They typically appear as T2 hyposignal lesions with nonspecific enhancement.

The choice of treatment depends on factors such as the patient's age, general health, primary tumor site, extent of metastasis, lesion size, rate of disease progression, and symptom severity. Treatment options can be either localized or systemic; however, it is important to consider that pRCC exhibits variable growth patterns. Resection of oligometastatic disease remains a standard approach, particularly in cases like this one, where the interval between metastases is long and the lesion is isolated.

According to the European Association of Urology Guidelines, complete surgical resection of synchronous or metachronous hematogenous metastases in metastatic RCC is the gold standard. Complete resection can achieve a 5-year tumor-specific survival rate of 49.4% [2, 10].

Due to the rarity of penile secondary malignancies, there are no specific treatment recommendations. Generally, the prognosis for patients with penile metastasis is poor due to widespread disease and poor general health, resulting in short survival. In our case, following the complete resection of a single metastasis from RCC, a good prognosis was expected and confirmed by this 7-year follow-up.

## 4. Conclusion

Penile metastases are rare and often indicate widespread disease with poor prognosis. However, in selected cases of oligometastatic spread, early detection and surgical resection may offer good long-term oncological outcomes. Management should be individualized based on patient condition, tumor origin, and extent of disease.

## Figures and Tables

**Table 1:** Immunohistochemistry marker.

**Marker**	**Results**	
TFE3	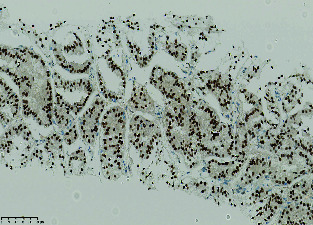	Nuclear immunostaining diffusely positive for TFE3
Ck broad spectrum	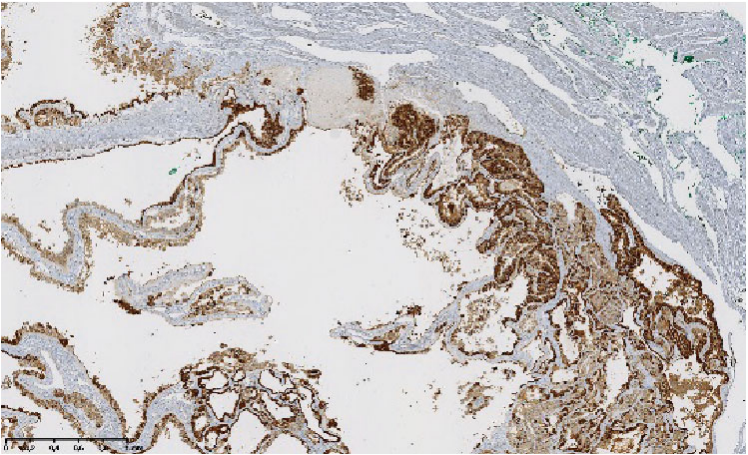	Confirming an epithelial tumor
PAX 8	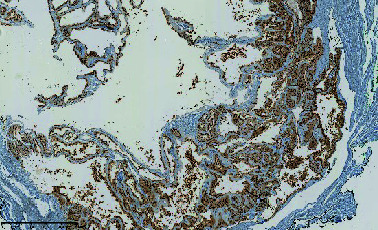	Diffusely positive. 90%–95% of renal cell carcinomas are PAX8-positive, irrespective of the subtype (clear cell, papillary, etc.). PAX8 is also positive in some other tumor types, e.g. thyroid carcinoma, but HE is not a picture of thyroid. PAX8 is used to screen for renal origin, irrespective of subtype, because each type has its own profile.
AMACR	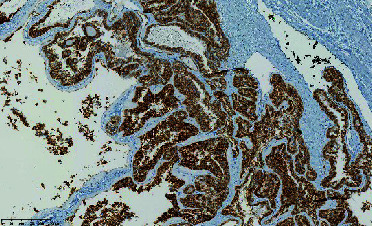	Diffusely positive, confirming a papillary type.
CD10	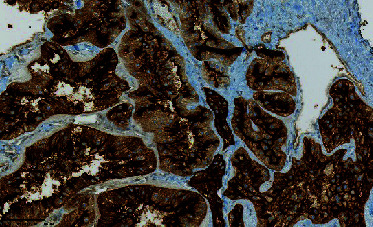	Apical membranous positivity; this can be seen in papillary type. Clear cell renal cell carcinoma (can be eosinophilic when high grade) shows circumferential membranous staining.
Ck7	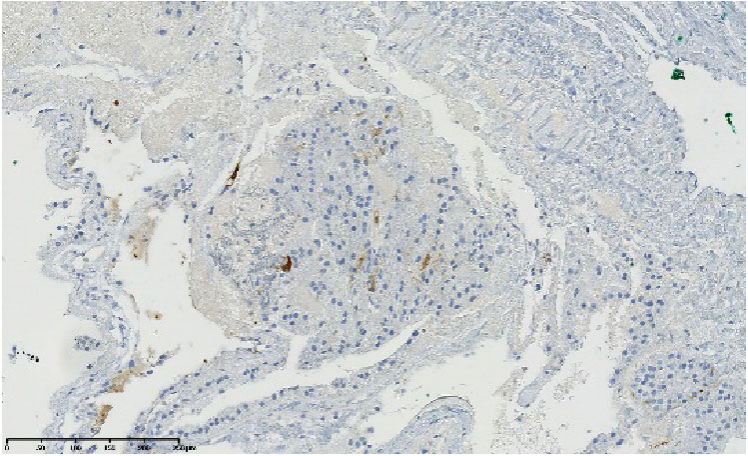	Very focally positive; Type 1 papillary renal are usually diffusely positive, Type 2 show much less, focal positivity.
TFE3	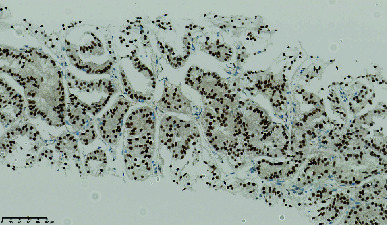	Nuclear immunostaining diffusely positive for TFE3

## Data Availability

The data supporting the findings of this study are available from the corresponding author upon reasonable request and with the authorization of the patient.
